# Does introducing an immunization package of services for migrant children improve the coverage, service quality and understanding? An evidence from an intervention study among 1548 migrant children in eastern China

**DOI:** 10.1186/s12889-015-1998-5

**Published:** 2015-07-15

**Authors:** Yu Hu, Shuying Luo, Xuewen Tang, Linqiao Lou, Yaping Chen, Jing Guo, Bing Zhang

**Affiliations:** Zhejiang Center for Disease Control and Prevention, Institute of Immunization and Prevention, No. 3399 Binsheng Road, Binjiang District, Hangzhou, P. R. China; Yiwu Center for Disease Control and Prevention, Institute of Immunization and Prevention, Yiwu, China

**Keywords:** Migrant children, Vaccination coverage, Interventions, EPI, Evaluation

## Abstract

**Background:**

An EPI (Expanded Program on Immunization) intervention package was implemented from October 2011 to May 2014 among migrant children in Yiwu, east China. This study aimed to evaluate its impacts on vaccination coverage, maternal understanding of EPI and the local immunization service performance.

**Methods:**

A pre- and post-test design was used. The EPI intervention package included: (1) extending the EPI service time and increasing the frequency of vaccination service; (2) training program for vaccinators; (3) developing a screening tool to identify vaccination demands among migrant clinic attendants; (4) Social mobilization for immunization. Data were obtained from random sampling investigations, vaccination service statistics and qualitative interviews with vaccinators and mothers of migrant children. The analysis of quantitative data was based on a “before and after” evaluation and qualitative data were analyzed using content analysis.

**Results:**

The immunization registration (records kept by immunization clinics) rate increased from 87.4 to 91.9 % (*P* = 0.016) after implementation of the EPI intervention package and the EPI card holding (EPI card kept by caregivers) rate increased from 90.9 to 95.6 % (*P* = 0.003). The coverage of fully immunized increased from 71.5 to 88.6 % for migrant children aged 1–4 years (*P* < 0.001) and increased from 42.2 to 80.5 % for migrant children aged 2–4 years (*P* < 0.001). The correct response rates on valid doses and management of adverse events among vaccinators were over 90 % after training. The correct response rates on immunization among mothers of migrant children were 86.8–99.3 % after interventions.

**Conclusion:**

Our study showed a substantial improvement in vaccination coverage among migrant children in Yiwu after implementation of the EPI intervention package. Further studies are needed to evaluate the cost-effectiveness of the interventions, to identify individual interventions that make the biggest contribution to coverage, and to examine the sustainability of the interventions within the existing vaccination service delivery system in a larger scale settings or in a longer term.

**Electronic supplementary material:**

The online version of this article (doi:10.1186/s12889-015-1998-5) contains supplementary material, which is available to authorized users.

## Background

Immunization is regarded as one of the remarkable public health achievements of the 20^th^ century and one of the most cost-effective public health service [1, 2]. Immunization have substantially reduced the burden of vaccine preventable diseases (VPDs) worldwide. China started Expanded Program on Immunization (EPI) in 1978 with four vaccines. Today, this program continues with eleven vaccines (Table 1). Every child should take one dose of Bacille Calmette-Guérin Vaccine (BCG), four doses of Poliovirus Vaccine (PV), four doses of Diphtheria-Pertussis-Tetanus Vaccine (DPT), three doses of Hepatitis B Vaccine (Hep B), two doses of Measles Containing Vaccine (MCV), two doses of Japanese Encephalitis Vaccine (JEV), one dose of Hepatitis A Vaccine(HepA), two doses of Meningococcal Polysaccharide Vaccine-type A(MPV-A), two doses of MPV-type A and C(MPV-AC), and one dose of Diphtheria-Tetanus Vaccine (DT) before the 7^th^ birthday [3]. Within Chinese vaccination service system, all the vaccines are administered through immunization clinics by vaccinators who have been trained. Routine vaccination is free of charge for every child including migrant children. Specifically, vaccines for routine EPI are financed by central government of China and other relative logistic supports (e.g. disposal syringe, cold chain maintenance and transportation) are financed by local governments [4].

**Table 1:**
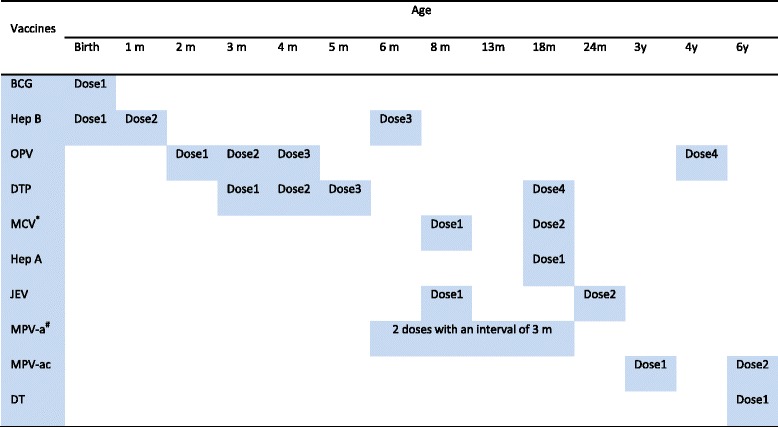
Recommended immunization schedule of Expanded Program on Immunization in China

*the 1st dose of MCV use Measles-rubella combined live attenuated vaccine and the 2nd dose of MCV use Measles-mumps combined live attenuated vaccine

^#^2 doses of MPV are scheduled from 6–18 months of age with an interval ≥3 months

The success of EPI depends on not only effective immunization schedules, but also high coverage rates. The lower coverage rates are common among migrant children due to the frequent mobility and the poor awareness of vaccination. As a developed city in east China, Yiwu has a total 1105.5 Km^2^ with a 2 million population. Yiwu’ s rapid social economic development, which is based on small commodity trade and vibrant free markets has attracted 1 million migrants (2014 census). Our previous study [5] indicated that the “fully immunized” coverage for vaccines scheduled before 12 months of age was 62.4 % among migrant children in Yiwu in 2011. Furthermore, 11.4 % of the surveyed migrant children received no immunizations (left-out), and the rest of them did not complete all vaccinations (drop-out) or received vaccinations too close together or at an earlier age (invalid doses) [5]. In a comprehensive review of vaccination service in developing countries [6], the authors pointed out some challenges that were not faced in the past. These challenges included the rapid population growth, the need for giving high priority to other recently emergent public health issues, and the need to use appropriate strategies to reach marginal population. Other barriers to achieving optimal coverage among migrant children included caregivers’ misinformation on immunization and poor awareness of completing the entire series, irregular opening time of immunization clinics, and poor accessibility among socio-economically disadvantaged migrants [7, 8].

Evidence from different areas have indicated that interventions can effectively improve vaccination coverage [9–12]. Coverage rates had been improved through strengthening the routine immunization service system and social mobilization, modification of the EPI session schedule, recruiting community volunteers actively participated with health providers in child-to-child search, developing a screening tool to identify unmet demand for vaccination, training of vaccinators.

The objective of our study was to evaluate the influence of the EPI intervention package in improving migrant children’s vaccination coverage in Yiwu, within the existing vaccination service delivery system. The EPI intervention package consisted of: (1) extending the EPI service time and increasing the frequency of vaccination service; (2) training for vaccinators on valid doses and management of adverse events; (3) developing a screening tool to identify vaccination demands among migrant clinic attendants; (4) social mobilization for immunization.

## Methods

### Study design and intervention package

Our study was an intervention trail with a pre- and post-test design. The intervention package was conducted for 32 months from October 2011 to May 2014. A pre-intervention investigation was conducted in September 2011 and a post-intervention investigation in June 2014.

Yiwu comprises 13 towns and each town has a health facility. Routine immunization services were provided by fixed immunization clinic in each health facility (outreach service was not allowed since 1998). All the 13 towns were selected as the unit of intervention, namely, the interventions were implemented throughout the entire town.

The EPI intervention package implemented in this study included following aspects:

1. Extending the EPI service time and increasing the frequency of vaccination service: the EPI service was provided from 8:00 am to 11:00 am once per week (on Wednesday) in every immunization clinics before this study. During the study, the service time of immunization clinics and the frequency of service were extended to allow migrant caregivers more time to attend.
2. Training program for vaccinators: a two days refreshing training was provided to all the vaccinators from 13 immunization clinics (107 vaccinators in total) in October 2011. The training program was designed by expert panel on immunization of Zhejiang provincial center for disease control and prevention (CDC). The training content focused on valid doses and management of Adverse Event Following Immunization (AEFI). A combination of training methods including lecture, group discussion, PowerPoint presentation and role-paly was applied. All the vaccinators completed the pre- and post- training questionnaires (see the Additional file 1: Table s1) to evaluate changes on immunization knowledge.
3. Developing a screening tool (see the Additional file 1: Figure s1) to identify vaccination demands among migrant clinic attendants: a screening tool was developed by Zhejiang provincial CDC and Yiwu CDC to check the immunization status of migrant children when their mothers visited a health facility for child care service or other medical service. If a migrant child required vaccination, it was provided immediately at the immunization clinic if available, otherwise, the child was appointed to the next nearest vaccination session. This intervention was implemented by the general practitioners in health facilities.
4. Social mobilization for immunization: Yiwu CDC formed social mobilization teams for each town, which consisted of general practitioner in health facilities, rental house owners, caregivers of fully immunized children, teachers of daycare centers/kindergartens/primary schools. The responsibilities of these teams were to assist in ensuring the registration of the EPI target migrant children, to disseminate EPI knowledge to migrants through booklets or pictorial cards, and to mobilize migrants to participate in the vaccination on their own initiatives. A half day orientation, led by expert panel on immunization of Zhejiang provincial CDC, was held for social mobilization team members. Monthly meetings were held with team members to review, praise and motivate their previous effort by Yiwu CDC.

### Study population

In this study, migrants referred to people living in an area without a household registration card issued by public security bureau of their current living areas. We determined the immigration status of children or their caregivers by checking their Resident ID Card or asking their residence address. The study population included migrant children aged 1–4 years (children born from 1 September 2007 to 31 August 2010 were selected for the pre-intervention investigation and children born from 1 June 2010 to 31 May 2013 were selected for the post-intervention investigation) and their mothers, vaccinators from 13 immunization clinics. Migrant children who had lived in the surveyed areas continuously for more than one month were included in this study. We documented the date of the last immigration of the surveyed migrant children, then we calculated the length of time for continuously living in the surveyed areas.

### Sampling

First, the proportion of migrant people of each villages or communities were calculated based on the data provided by Bureau of Statistics of Yiwu. Second, villages or communities where migrants accounted for more than half of the total population were selected as investigation sites. Finally, 56 investigation sites for the pre-intervention investigation and 57 investigation sites for the post-intervention investigation were selected, respectively. Second, we calculated the sample size by the method of simple random sampling [13], setting an assumption coverage of 85 %, a significance level of 0.05, a design effect level of 2 and a desired precision of 3 % for the pre-investigation. For the post-investigation, the desired precision increased into 5 %. The final sample size were 1120 (20 children per cluster for 56 clusters) and 399 (7 children per cluster for 57 clusters) for the pre- and post-investigation, respectively.

### Data collection

Data were obtained from three sources: (1) random sample investigation to evaluate the vaccination coverage, (2) vaccination service statistics of immunization clinics, and (3) qualitative components from vaccinators and mothers of migrant children. These data were collected before and after the implementation of the EPI intervention package. Supervisors from Zhejiang provincial CDC were required to review all these data for logistic errors or blanks. All these errors should be addressed through reinvestigation when needed.

#### Random sampling investigation

Both the pre- and the post- intervention investigation were implemented in 2 weeks before and after the intervention period immediately. We got household list of every investigation site from local administrative office and used the random number to select one household as the first one to be investigated. If there was more than one eligible child in a household, the child whose birthday was the closest to the survey day was investigated. After found the first eligible child, we continued choosing subsequent household located at the right of the previous one until all the eligible children for each investigation site were investigated. A standardized, pretested questionnaire (see the Additional file 1: Table s2) was used for face-to-face interviews for both the pre- and the post- intervention investigation. Demographic details, such as child gender, number of children in family, mother’s age, mother’s education level, child’s birth place and monthly household income per capita, and mother’s knowledge on immunization were collected. Children’s vaccination status were confirmed by checking the immunization card kept by caregivers or immunization records kept by immunization clinics. Only written vaccination records were applied to avoid inaccuracies or recall bias. Twenty-six staff of Yiwu CDC were selected and trained as interviewers in this study. A training meeting was held for all the interviewers to ensure they were familiar with the questionnaire and investigation skill for sensitive question. Public health liaisons (some private doctors recruited by village level administration) of each investigation site were invited as guiders for help.

#### Vaccination service statistics

Vaccination service statistics (the EPI service time, the frequency of the vaccination service, number of attendance of the vaccination session) were collected from each immunization clinic and reviewed to ascertain the changes in performance of vaccination service. The effect of training was assessed through the changes in the incidence of invalid doses/AEFI among migrant children, and the knowledge level on immunization of vaccinators. Data on using the screening tool (number screened for immunization demands, number of demands identified, number followed up to ascertain its outcome) were also collected. Furthermore, data or field notes on the number of notification for registration or vaccination, social mobilization for immunization were also documented. All these data were collected by staff of Yiwu CDC.

#### Quality components from mothers of migrant children and vaccinators

Experts on immunization from Zhejiang provincial CDC organized a group discussion with mothers of migrant children. The mothers whose children were fully immunized in the post-intervention investigation were randomly selected. The discussion focused on the reasons for fully immunization, timely vaccination, and challenges they faced in existing vaccination service delivery system. Experts on immunization from Zhejiang provincial CDC also held a review meeting with 13 vaccinators (one vaccinator from each immunization clinic) after intervention to collect their perceptions on the EPI intervention package, and challenges faced in implementation.

### Data analysis

Quantitative data were analyzed with the SPSS version 13.0 software. The analysis of quantitative data was based on a “before and after” evaluation. Pearson *χ*^*2*^ test was adopted to compare the difference in specific vaccination coverage and mothers’ demographic categorical variables before and after the implementation of the EPI intervention package. A *P* < 0.05 was considered to be significant. Qualitative data collected through observation, group discussion and review meeting were first documented, then coded, categorized and abstracted manually by carefully reading field notes, and finally analyzed using content analysis.

### Ethics statement

This study was approved by the Ethical Review Board of Zhejiang Provincial Center for Disease Control and Prevention. In each random sampling survey, the informed consent form on behalf of children and their caregivers enrolled was discussed with children’ s caregivers, and signed by one of them once there was a decision to participate.

## Results

### Vaccination coverage survey among migrant children aged 1–4 years

#### Social demographic characteristics

A total of 1136 migrant children and 412 migrant aged 1–4 years were enrolled in the pre-intervention investigation and the post-intervention investigation, respectively. The social demographic characteristics of migrant children and their mothers investigated before and after the intervention did not differ significantly, which indicated that no heterogeneity was found between two samples (Table 2).

**Table 2:** Social demographic characteristics of migrant children aged 1–4 years and their mothers before and after the implementation of EPI intervention package in Yiwu

Variables	Before interventions (N = 1136)		After interventions (N = 412)		*χ* ^*2*^	*P*
	N	%	N	%		
Maternal age					3.533	0.060
< 30 y	716	63.0	281	68.2		
≥ 30y	420	37.0	131	31.8		
Maternal education level					5.844	0.211
Illiteracy	15	1.3	3	0.7		
Primary school	78	6.9	26	6.3		
Junior middle school	620	54.6	242	58.7		
Senior middle school	315	27.7	94	22.8		
College or above	108	9.5	47	11.4		
Mothers working time					1.612	0.447
No job	240	21.1	82	19.9		
≤ 8 h	213	18.8	89	21.6		
> 8 h	683	60.1	241	58.5		
Gender of child					0.247	0.619
Male	598	52.6	211	51.2		
Female	538	47.4	201	48.8		
Age of child					1.644	0.440
1 year-	524	46.1	204	49.5		
2 years-	415	36.5	145	35.2		
3–4 years	197	17.3	63	15.3		
Number of children in family					0.947	0.623
1	445	39.2	171	41.5		
2	608	53.5	209	50.7		
≥ 3	83	7.3	32	7.8		
Birth place of child					0.655	0.418
Hospital	1024	90.1	377	91.5		
At home	112	9.9	35	8.5		
Latest continuously living time of child					1.606	0.448
3–6 months	266	23.4	84	20.4		
7–12 months	479	42.2	179	43.4		
≥ 13 months	391	34.4	149	36.2		
Monthly household income per capita					0.698	0.705
< 800 CNY	208	18.4	68	16.5		
800–1500 CNY	589	51.8	220	53.4		
> 1500 CNY	339	29.8	124	30.1		

#### Vaccination coverage

The immunization registration (records kept by immunization clinics) rate and the EPI card holding (EPI card kept by caregivers) rate improved significantly after the implementation of the EPI intervention package. The immunization registration rate increased from 90.3 to 96.6 % (*χ*^*2*^ *=* 5.124*, P =* 0.024) and the EPI card holding rate increased from 93.6 to 96.6 % (*χ*^*2*^ *=* 6.511*, P =* 0.011) among migrant children who were continuously living in the investigation sites for at least 13 months. The left-out rate (based on EPI cards) decreased from 9.1 to 4.4 % (*χ*^*2*^ *=* 8.621*, P =* 0.003) (Table 3). The coverage of primary vaccination improved for all the recommended antigens and the drop-out rate decreased from 28.5 to 11.4 % (*χ*^*2*^ *=* 47.658*,P* < 0.001) (Table 4). The timeliness of the first dose of MCV increased from 42.7 to 89.3 % (*χ*^*2*^ *=* 263.813, *P* < 0.001). The coverage of boost vaccination among migrant children aged 2–4 years increased for all the recommended antigens and the drop-out rate decreased from 57.8 to 19.5 % (*χ*^*2*^ *=* 105.579, *P* < 0.001) (Table 5).

**Table 3:** Immunization registration rate and EPI card holding rate among migrant children aged 1–4 years before and after the implementation of EPI intervention package in Yiwu

Variables		Immunization registration rate before/after intervention				EPI card holding rate before/after intervention			
		Before (n/N, %)	After (n/N, %)	*χ* ^*2*^	*P*	Before (n/N, %)	After (n/N, %)	*χ* ^*2*^	*P*
Age of child	1 year-	472/524, 90.1	190/204, 93.1	1.318	0.251	490/524, 93.5	197/204, 96.5	2.039	0.153
	2 years-	359/415, 86.5	133/145, 91.7	2.275	0.132	372/415, 89.6	139/145, 95.8	4.462	0.035
	3–4 years	162/197, 82.2	56/63, 88.9	1.108	0.292	171/197, 86.8	58/63, 92.1	0.807	0.369
Latest continuously living time of child	3–6 months	210/266, 78.9	71/84, 84.5	0.927	0.336	229/266, 86.1	77/84, 91.7	1.335	0.248
	7–12 months	430/479, 89.8	164/179, 91.6	0.319	0.572	438/479, 91.4	169/179, 94.4	1.222	0.269
	≥13 months	353/391, 90.3	144/149, 96.6	5.124	0.024	366/391, 93.6	148/149, 96.6	6.511	0.011
Total		993/1136, 87.4	379/412, 91.9	5.843	0.016	1033/1136, 90.9	394/412, 95.6	8.621	0.003

**Table 4:** Primary vaccination coverage of migrant children aged 1–4 years before and after the implementation of EPI intervention package in Yiwu

Vaccines	Dose	Coverage before interventions (N = 1136)		Coverage after interventions (N = 412)		*χ* ^*2*^	*P*
		N	%	N	%		
BCG		1021	89.9	388	94.2	6.318	0.012
Hep B	1	1038	91.4	406	98.6	23.674	<0.001
	2	1030	90.7	401	97.3	18.259	<0.001
	3	1001	88.1	396	96.2	21.084	<0.001
PV	1	1044	91.9	406	98.5	21.389	<0.001
	2	1038	91.4	403	97.7	18.514	<0.001
	3	1022	90.0	397	96.3	15.358	<0.001
MCV	1	982	86.4	409	99.3	53.196	<0.001
Timeliness^a^		485	42.7	368	89.3	263.813	<0.001
DPT	1	1037	91.3	405	98.3	22.244	<0.001
	2	1027	90.4	400	97.2	17.821	<0.001
	3	1010	88.9	395	95.9	16.674	<0.001
JEV-Li	1	966	85.0	399	96.9	39.325	<0.001
Fully immunized^#^		812	71.5	365	88.6	47.658	<0.001

^a^: Timeliness of MCV_1_ was defined as MCV vaccination occurring within the first day to the final day of the 8^th^ month of life. ^#^: Fully immunized was defined by receiving one dose of BCG, one dose of MCV, one dose of JEV, 3 doses of Hep B, 3 doses of DPT and 3 doses of PV

**Table 5:** Booster vaccination coverage of migrant children aged 2–4 years before and after the implementation of EPI intervention package in Yiwu

Vaccines	Dose	Coverage before interventions (N = 612)		Coverage after interventions (N = 208)		*χ* ^*2*^	*P*
		N	%	N	%		
Men-A	1	529	86.4	205	98.4	23.016	<0.001
	2	472	77.1	199	95.9	34.685	<0.001
MCV	2	335	54.8	200	96.2	104.515	<0.001
DPT	4	331	54.1	195	93.7	104.479	<0.001
Hep A		320	52.3	200	96.2	126.872	<0.001
Fully immunized^#^		258	42.2	167	80.5	105.579	<0.001

^#^: Fully immunized was defined by receiving one dose of BCG, 2 doses of MCV, one dose of JEV, one dose of Hep A, 2 doses of Men-A, 3 doses of Hep B, 4 doses of DPT and 3 doses of PV

### Performance of EPI intervention package

1. Extending the EPI service time and increasing the frequency of vaccination service

**Fig. 1:**
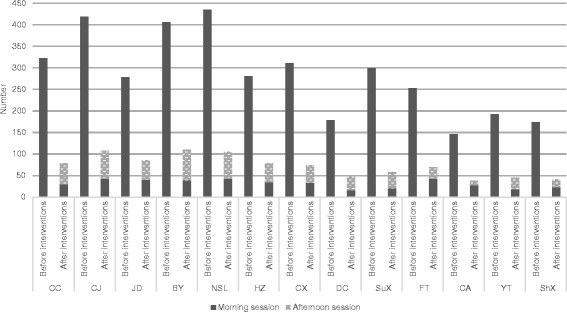
Number of migrant children immunized on the vaccination sessions before and after the implementation of EPI intervention package in Yiwu. Notes: CC: Choucheng, CJ: Choujiang, JD: Jiangdong, BY: Beiyuan, NSL: Niansanli, HZ: Houzhai, CX: Chengxi, DC: Dacheng, SuX: Suxi, FT: Fotang, CA: Chian, YT: Yiting, ShX: Shangxi

During the intervention period, all the immunization clinics added an afternoon session from 13:00 to 16:00. More children were immunized in the afternoon sessions than during the morning sessions. Another change was all the immunization clinics increased the frequency of vaccination service from once per week to 4 times per week. Furthermore, 10 immunization clinics scheduled the vaccination sessions on Saturday or Sunday. The number of children received vaccination for each session was also declined after implementation of this intervention (Fig. 1).

The qualitative data showed that the mothers highly appreciated this modification of the EPI session schedule. Three mothers who attended the group discussion said, “it would not have been possible for us to complete all the doses if these changes did not happen”. The vaccinators reported that this intervention was one of the main impact factors for the improvement of vaccination coverage. They stated that migrant caregivers could bring their children to the immunization clinics whenever they were available as the service providers stayed for a longer time. Besides, they added that the caregivers with no jobs could also vaccinate their children at a convenient time as the service delivery period was extended.

(2) Training program for vaccinators

The correct response rates on valid doses and management of AEFI of vaccinators were more than 90 % after training, compared with 32.7–86.9 % before training (*P* < 0.001 for all terms) (Table 6). In terms of practice, not a single invalid dose was found after training, while 16.2 % of the investigated migrant children had invalid doses before training. Pyogenic abscess among migrant children after training were less than 0.5 % compared with 1.5 % before training (*χ*^*2*^ *=* 45.381, *P* < 0.001). The vaccinators stated that the training helped them in reducing invalid doses and improving the capacity in managing the AEFI. Furthermore, they also paid more attention to the health screening before immunization.

**Table 6:** Knowledge level of vaccinators on immunization before and after a two days refreshing training

Questions	Correct response rate (n/%)		*χ* ^*2*^	*P*
	Before interventions (N = 107)	After interventions (N = 107)		
The recommended age of the first dose of MCV was 8^th^ months	93/86.9	102/95.3	125.789	<0.001
The interval time between the 3rd dose of DPT and the 4^th^ dose DPT is 6 months	68/63.6	104/97.2	45.712	<0.001
The minimum interval of two live parenteral vaccines is 28 days or four weeks	84/78.5	105/98.1	90.770	<0.001
Live attenuated vaccines and inactive vaccines can be administered simultaneously at the same visit	59/55.1	104/97.2	32.969	<0.001
The minimum interval between the two doses of Men-A is 3 months	78/72.9	106/99.1	72.083	<0.001
The majority of AEFI are mild and self-limited	64/59.8	103/96.3	37.957	<0.001
Adverse reactions following live vaccines usually occur 7–21 days after the vaccine was given	35/32.7	98/91.6	29.094	<0.001
All the AEFIs need to report to the online National AEFI Surveillance System	82/76.6	106/99.1	84.857	<0.001
The risk of AEFI could be minimized by rigorous screening	90/84.1	107/100.0	117.520	<0.001
One of the first aid procedures of an acute anaphylactic shock is Injection of norepinephrine or epinephrine	85/79.4	104/97.2	93.335	<0.001

(3) Developing a screening tool to identify vaccination demands among migrant clinic attendants

Totally, 15,279 mothers were screened. 2984 children (19.5 %) were identified as having unmet demands for immunization. Of these 2984 migrant children, 2104 children (70.5 %) were appointed to the next nearest vaccination session and the rest of them were vaccinated immediately when they were identified. All the identified migrant children were followed by the vaccinators till they received the vaccination. The vaccinators stated that the screening tool helped them reduce the drop-outs and they suggested it be introduced to the public health liaisons in communities or villages for further improvement on vaccination coverage. They also stated that the tool was easy to use and it required only 3 min on average for screening one child.

(4) Social mobilization among migrants

Although 87.5 % (20 monthly meetings of the planned 32) of the monthly meetings formed, all the social mobilization team members attended each of these meetings. The records of the team members showed that they identified 172 left-outs and 458 drop-outs during the study period, respectively. Mothers of these migrant children were motived, followed up until their children were vaccinated.

The knowledge level on immunization of mothers of migrant children improved after intervention (Table 7). Mothers of migrant children stated that the social mobilization team members had visited them several times and motived them to immunize their children. Besides, they were informed on the benefits of full immunization and timely immunization.

**Table 7:** Knowledge level of migrant mothers on immunization before and after the interventions

Knowledge on immunization	Correct response rate (n/%)		*χ* ^*2*^	*P*
	Before interventions (N = 1136)	After interventions (N = 412)		
Know the vaccination was necessary	1055/92.9	409/99.3	22.916	<0.001
Know the immunization schedule	841/74.0	377/91.5	53.997	<0.001
Immunity can be achieved without vaccination	945/83.2	372/90.2	11.468	0.001
Vaccine was effective	1033/90.9	394/95.6	8.621	0.003
The side-effects of vaccination are usually not serious	986/86.8	386/93.6	13.582	<0.001
Postponed vaccination increase the susceptible period of children	913/80.4	358/86.8	8.319	0.004
Vaccination was costly	1027/90.4	405/98.4	26.066	<0.001
Only fully immunized for a specific antigen could be protectable	993/87.4	379/91.9	5.843	0.016

The vaccinators felt that the social mobilization teams palyed a key role in reminding migrants about vaccination. As mothers of migrant children always forgot the due dates of doses, the social mobilization teams could remind mothers about the date of a due dose and inform the vaccination session day through visiting their family. The vaccinators also suggested that the public health liaisons or other volunteer from communities or villages should be better able to do social mobilization because they lived in close to these migrant people and had acute and regular information on pregnant women, newborns, which children were vaccinated and which were not.

## Discussion

This study indicated that implementation of the EPI intervention package as a part of the existing EPI service delivery system had improved the vaccination coverage among migrant children in Yiwu. Proportion of fully immunized children had increased, and the drop-outs and the proportion of invalid doses had declined dramatically. Those improvements showed a positive overall influence of this EPI intervention package.

Although the issue of overlapping effects of the different interventions could not be avoided in this study and the aims of this study did not include the assessment of individual contributions to the overall impact on immunization service of migrant children, the process indicators and qualitative data analysis confirmed the potential contributions of the individual interventions. More children were vaccinated in the afternoon sessions than in the morning sessions indicated that the extended EPI service time was popular with mothers of migrant children. This finding was echoed by mothers of migrant children and vaccinators through group discussion. It was also consistent with the WHO recommendation that the working time of immunization clinics should be extended [14, 15]. The extended service time of vaccination session may be most beneficial to the working mothers whose children had a higher possibility of drop-out or left-out. In this study, almost 80 % of migrant women in childbearing age had jobs, hence, this intervention may improve the mothers’ compliance with the immunization schedule and allow for a better coverage. The EPI service time of all 13 immunization clinics was extended up to 16:00 pm during the intervention period. Initially, it was suggested that the EPI service time be extended up to 17:00 pm, but it was considered infeasible by the vaccinators because each child needed a 30 min observation after vaccination and all the vaccinators knocked off at 16:30 pm. The two days training was probably the driving force for reducing invalid doses and improving the knowledge level on management of AEFI. This inference was probably true as most vaccinators lacked clear concepts of valid doses and management of AEFI in Yiwu and previous studies reported the success of training in solving these problems [16–18]. The utilization of the screening tool was effective in identifying and addressing the unmet demands. However, the benefits needed to be weighed against the costs of this intervention as only a small part of migrant children could be identified through this mechanism. For example, the screening tool was implemented only in health facilities, it was likely that some drop-outs who did not seek medical care from health facilities could not be addressed. As suggested by the vaccinators, expanding this intervention to public health liaisons in communities or villages could broaden the reach of this strategy and make it more effective. Since the community bond in migrants were not as strong as that in residents, the role of social mobilization team was likely to be particularly important. Consistently with previous reports [19–21], social mobilization improved the maternal compliance with immunization and knowledge level on immunization in this study. Although it would have overlapping effects from other interventions in increasing coverage, the social mobilization team actually played a role of channeling which was demonstrated to be one of the effective strategies for improving the vaccination coverage [21].

Beyond the effectiveness of this EPI intervention package mentioned above, the sustainability was another key issue. Although we did not evaluate the sustainability formally, a number of evidence indicated that this EPI intervention package was sustainable. First, the EPI intervention package was implemented within the existing vaccination service delivery system. Yiwu CDC and the 13 immunization clinics were all well motived to improve vaccination services for migrant children. The fact that migrant children had been considered as a week point and high priority of EPI in Yiwu, coupled with the national focus on the measles elimination activity, was the main reasons for the motivation on the part of the Yiwu CDC level. On the other hand, the logistic supports of EPI financed by local government were allocated by Yiwu CDC, which encouraged the immunization clinics to involve in the study and make improvements in vaccination service for migrant children. Second, in terms of implementation, the organizations and individuals involved integrated all the interventions into their ongoing programs without any extra cost. For example, vaccinators from the 13 immunization clinics were willing to extend the EPI service time and increase the frequency of vaccination service without additional salaries. The general practitioners in health facilities implemented the screening tool as a part of their ongoing work without any complain. We thought the training program for vaccinators was sustainable, because training for vaccinators was already a routine program of Yiwu CDC and the training materials on valid doses and management of AEFI had already been developed. Furthermore, if we find the needs of improving other knowledge and practice on vaccination, the mechanism of this intervention including training, assessment, and supervision can also be applied. Third, although social mobilization team members seemed interest to work throughout the intervention period, they were not willing to maintain the current activity. The main reason was that they had no financial payments to participate. In fact, the effectiveness of this intervention varied in different areas. The rates and frequencies of immigration into and out of towns would affect the capacity and willingness of social mobilization team members to participate over time.

This study was subjected to several limitations. First, this study was not a control design and it restricted the conclusions that could be drawn based on these results. However, the majority (52, 92.9 %) of investigation sites in the pre-intervention investigation retained in the post-intervention investigation and implementing the investigations in the same areas would have strengthened the design. The results were homogenous in terms of the social demographic characteristics of migrant children and their mothers, which suggested that this should not have confounded the results. Actually, the wide diversity among villages and communities in terms of variables such as demographic characteristics of migrant people, local health service system, migrant population size, made it difficult to match a control. Second, theoretically, there were confounders from other factors to improve coverage, but there was no evidence of other interventions in Yiwu during the study period. Third, the cost-effectiveness analysis on this EPI intervention package was not conducted, which made it difficult to determine whether these interventions are sustainable in a longer term or in some resources limited settings.

## Conclusion

Our study showed a substantial improvement in vaccination coverage among migrant children in Yiwu after implementation of the EPI intervention package. This package included both demand-side and supply-side interventions appeared to lead to an ideal vaccination coverage among migrant children aged 1–4 years. All of these interventions showed effective, especially for the extending the EPI service time and increasing the frequency of vaccination service, but the screening tool to identify vaccination demands among migrant clinic attendants only covered a small proportion of target population, indicating it may not be as cost-effective as other interventions. This EPI intervention package was sustainable as it was implemented within the existing public health service system and did not need additional resources. Further studies are needed to evaluate the cost-effectiveness of the package, to identify individual interventions that make the biggest contribution to coverage, and to examine the sustainability of the package within the existing vaccination service delivery system in a larger scale settings or in a longer term.

## Additional file

Additional file 1: Table s1. Knowledge Level on Valid Immunization and Management of AEFI of Vaccinators. **Figure s1.** Screening tool for identifying the demands among migrant children. **Table s2.** Questionnaire for Migrant Children and Their Mother on Vaccination Service and Knowledge (for Evaluation of pre- and post-Intervention).
