# Supplementary material for: Does introducing an immunization package of services for migrant children improve the coverage, service quality and understanding? An evidence from an intervention study among 1548 migrant children in eastern China
Source: BMC Public Health. 2015 Jul 15;15:664. doi: 10.1186/s12889-015-1998-5 (PMC4501193; doi:10.1186/s12889-015-1998-5)
Supplement: Additional file 1: Table s1. — Knowledge Level on Valid Immunization and Management of AEFI of Vaccinators. Figure s1. Screening tool for identifying the demands among migrant children. Table s2. Questionnaire for Migrant Children and Their Mother on Vaccination Service and Knowledge (for Evaluation of pre- and post-Intervention). [file 12889_2015_1998_MOESM1_ESM.docx]

Supporting information file:

**Table s1. Knowledge Level on Valid Immunization and Management of AEFI of Vaccinators**

Name of vaccinators: Date of evaluation:

Round of evaluation: (1)before training (2)after training □

| Questions | Right | False | Not sure |
| --- | --- | --- | --- |
| 1.The recommended age of the first dose of MCV was 8th months |  |  |  |
| 2.The interval time between the 3rd dose of DPT and the 4th dose DPT is 6 months |  |  |  |
| 3.The minimum interval of two live parenteral vaccines is 28 days or four weeks |  |  |  |
| 4.Live attenuated vaccines and inactive vaccines can be administered simultaneously at the same visit |  |  |  |
| 5.The minimum interval between the two doses of Men-A is 3 months |  |  |  |
| 6.The majority of AEFI are mild and self-limited |  |  |  |
| 7.Adverse reactions following live vaccines usually occur 7–21 days after the vaccine was given |  |  |  |
| 8.All the AEFIs need to report to the online National AEFI Surveillance System |  |  |  |
| 9.The risk of AEFI could be minimized by rigorous screening |  |  |  |
| 10.One of the first aid procedures of an acute anaphylactic shock is Injection of norepinephrine or epinephrine |  |  |  |

**Figure s1. Screening tool for identifying the demands among migrant children**

**Table s2 Questionnaire for Migrant Children and Their Mother on Vaccination Service and Knowledge (for Evaluation of pre- and post-Intervention)**

**Introductory note:**

For dear migrant child as well as mother: This following questionnaire is about migrant child’s immunization situation and its potential determinants. It will help us to improve the immunization service. Thank you for your participation.

**Part 1 Code for questionnaire**

| 1. Standard county No.: | □□□□□□ |
| --- | --- |
| 2. Villages/communities No.： | □□ |
| 3. Target child No.： | □□ |

**Part 2 Family and child’s information**

| 4. Name of surveyed child: _____________ | |
| --- | --- |
| 5. Mother’s name: _____________Cell phone：_____________ | |
| 6.Gender of the child: (1) Male (2) Female | □ |
| 7.Child’s birthday(yyyy/mm/dd): | □□□□□□□□ |
| 8.Mother’s age: (1) <30 (2)≥30 | □ |
| 9.Mother’s education level: (1) Illiteracy (2)Primary school (3)Junior middle school (4)Senior middle school (5)College or above | □ |
| 10. Mothers working time: (1)No job (2)≤8 hours (3)>8 hours | □ |
| 11. How many children do the family have? | □ |
| 12.Place of delivery(where did the child born?) : (1) Hospital (2) Home | □ |
| 13.When was the last immigration to the surveyed areas of child?  (yyyy/mm//dd): | □□□□□□□□ |
| 14. Household monthly income(CNY):(1)<800 (2)800-1500 (3)>1500 | □ |
| 15. Does child have an EPI card kept by caregivers? (1)Yes (2)No | □ |
| 16. Does child have a EPI record kept by immunization clinics (1)Yes (2)No | □ |

**Part 3 Vaccination status of migrant children**

| Vaccine | Dose | Vaccinated date (yyyy/mm/dd) |
| --- | --- | --- |
| BCG |  | □□□□□□□□ |
| Hep B | 1 | □□□□□□□□ |
|  | 2 | □□□□□□□□ |
|  | 3 | □□□□□□□□ |
| OPV | 1 | □□□□□□□□ |
|  | 2 | □□□□□□□□ |
|  | 3 | □□□□□□□□ |
| MCV | 1 | □□□□□□□□ |
|  | 2 | □□□□□□□□ |
| DPT | 1 | □□□□□□□□ |
|  | 2 | □□□□□□□□ |
|  | 3 | □□□□□□□□ |
|  | 4 | □□□□□□□□ |
| JEV-Li | 1 | □□□□□□□□ |
|  | 2 | □□□□□□□□ |
| Hep A |  | □□□□□□□□ |
| Men-A | 1 | □□□□□□□□ |
|  | 2 | □□□□□□□□ |
| Men-AC | 1 | □□□□□□□□ |

**Part 4 knowledge level on vaccination of migrant mothers**

| 17. Do you think immunization is necessary for your child?  (1)Yes (2)No (3)Not sure | □ | |
| --- | --- | --- |
| 18. Do you know the immunization schedule of your child?  (1)Yes (2)No | □ | |
| 19.What do you think that child’s immunity can be achieved without vaccination?  (1) Yes, that is right (2) No, that is not true (3) I don’t know | | □ |
| 20. Is vaccination effective for preventing diseases?  (1)Yes (2)No (3)Not sure | | □ |
| 21. Do you consider that the side-effects of vaccination are usually serious?  (1)Yes (2)No (3)Not sure | | □ |
| 22. Do you consider that postponed vaccination increases the suspectible period of children?  (1)Yes (2)No (3)Not sure | | □ |
| 23. Do you consider that vaccination is costly?  (1)Yes (2)No | | □ |
| 24. Do you consider that only fully immunized for a specific vaccine can be protectable?  (1)Yes (2)No (3)Not sure | | □ |

Interviewer Signature: ___________ Qulity control Signature： ___________

Signature Date: ___________ Signature Date: _____ ______
